# Socioeconomic and urban-rural inequalities in the population-level double burden of child malnutrition in the East and Southern African Region

**DOI:** 10.1371/journal.pgph.0000397

**Published:** 2023-04-25

**Authors:** Rishi Caleyachetty, Niraj S. Kumar, Hana Bekele, Semira Manaseki-Holland

**Affiliations:** 1 Warwick Centre for Global Health, Warwick Medical School, University of Warwick, Coventry, United Kingdom; 2 University College London Medical School, University College London, London, United Kingdom; 3 World Health Organization, Inter-Country Support Team, Zimbabwe WHO Country Office, Harare, Zimbabwe; 4 Institute for Applied Health Research, College of Medical and Dental Sciences, University of Birmingham, Birmingham, United Kingdom; Al-Bayan University, IRAQ

## Abstract

Socioeconomic and urban-rural inequalities in the population-level double burden of child malnutrition threatens global nutrition targets 2025, especially in East and Southern Africa. We aimed to quantify these inequalities from nationally representative household surveys in the East and Southern African region. 13 Demographic and Health Surveys between 2006 and 2018 including 72,231 children under five years old were studied. Prevalence of stunting, wasting and overweight (including obesity) were disaggregated by wealth quintiles, maternal education categories and urban-rural residence for visual inspection of inequalities. The slope index of inequality (SII) and the relative index of inequality (RII) were estimated for each country. Regional estimates of child malnutrition prevalence and socioeconomic and urban-rural inequalities were generated from pooling country-specific estimates using random-effects meta-analyses. Regional stunting and wasting prevalence were higher among children living in the poorest households, with mothers with the lowest educational level and in rural areas. In contrast, regional overweight (including obesity) prevalence was higher among children living in the richest households, with mothers with the highest educational level and urban areas. This study indicates pro-poor inequalities are present in child undernutrition and pro-rich inequalities are present in child overweight including obesity. These findings re-emphasise the need for an integrated approach to tackling the population-level double burden of child malnutrition in the region. Policy makers must target specific populations that are vulnerable to child malnutrition, to avoid further widening of socioeconomic and urban-rural inequalities.

## Introduction

With slow progress to tackle child undernutrition in Africa, the global health community has simultaneously seen a rapid rise in child overweight and obesity [1]. The African region has the highest burden of childhood stunting and one of the highest burdens of childhood overweight in Africa [2]. Importantly, the region is off track to achieve the UN Sustainable Development Goal (SDG) 2 aim to end all forms of hunger and malnutrition by 2030 [3]. The coexistence of child overnutrition (overweight and obesity) alongside undernutrition (stunting and wasting) has been termed the double burden of child malnutrition and is commonly assessed at the population level [4]. The double burden of child malnutrition is considered a major global health challenge for African countries [5], particularly in East and Southern Africa. Among children under five years of age at a population-level, prevalence of stunting is 34.5% in East Africa and overweight (including obesity) is 10.2% in Southern Africa. Overall, in Africa, prevalence of stunting is 29.1% and overweight (including obesity) is 4.7% [2].

Following on from the 2008 WHO Commission on Social Determinants of Health and 2011 Rio Political Declaration on Social Determinants of Health, another important goal for the African region is tackling socioeconomic inequalities in health [6]. Considering that the double burden of malnutrition is inextricably bound to socioeconomic conditions [7], monitoring and description of child malnutrition inequalities will be important to serve evidence-based program and policy decisions in the East and Southern African region. Several approaches to estimating socioeconomic inequality in health have been described [8]. These can include measures of association (e.g., frequency ratios, absolute differences in frequencies), measures of population impact (e.g., population attributable risk) and indices based on the ranking of the socioeconomic variable (e.g., slope and relative indices of inequality). The relative index of inequality and the slope index of inequality are the two major indices recommended for the measurement of socioeconomic inequalities in health, and quantify the socioeconomic gradient in relative and absolute terms, respectively [9]. In the context of child malnutrition, absolute inequality measures reflects the magnitude of difference in malnutrition prevalence between two subgroups and relative inequality measures show proportional differences in child malnutrition prevalence among subgroups. The choice of measure is not value-neutral and influences our understanding of which populations may be actually experiencing a higher malnutrition burden. Therefore, both relative and absolute measures to monitor socioeconomic inequalities are recommended [10].

The WHO African region has three inter-country support teams including for East and Southern Africa. In the East and Southern African region, there has not been to date a comprehensive assessment of socioeconomic inequalities in the population-level of the double burden of child malnutrition. Such an assessment would be valuable to support countries moving towards regional nutrition equity and achieve the World Health Assembly (WHA) and SDG nutrition related targets. We focus on 17 priority countries (Botswana, Comoros, Eswatini, Kenya, Lesotho, Madagascar, Malawi, Mauritius, Mozambique, Namibia, Rwanda, Seychelles, South Africa, Tanzania, Uganda, Zambia; Zimbabwe) as identified by the WHO Africa inter-country support team for East and Southern Africa (H. Bekele 2019, pers. comm., 25 Sept). Using nationally representative household surveys from East and Southern Africa conducted between 2006–2018 we quantified socioeconomic and urban-rural inequalities in the population-level double burden of malnutrition among children under five years old.

## Methods

We searched for surveys from the most recent Demographic and Health Survey (DHS) in every country identified as a priority country, which were conducted between 2006–2018, with data available for children under age five years living with their mother with valid weight and height measurements.

The DHS are nationally representative cross-sectional household surveys done at about 5-year intervals across East and Southern Africa. The surveys followed the same standardised procedures. Complete descriptions of country DHS sampling, questionnaire validation, data collection methods, and data validation procedures are published elsewhere [11]. The datasets used for this project are available at the DHS Program website (https://www.dhsprogram.com/data/available-datasets.cfm).

### Ethics statement

The DHS was approved centrally by ICF International (Calverton, MD, USA) institutional review board and by individual review boards within every participating country. Informed verbal consent was obtained from all participants during surveys and data were released in deidentified form. A parent or guardian provided verbal consent prior to participation by a child. This was a secondary analysis of data and, therefore, no further approval was required for this study. Further information about the DHS data usage and ethical standards are available at http://goo.gl/ny8T6X.

### Anthropometric measurements

DHS included data about each child’s age (in months and years) and measured height/weight. Trained survey staff weighed and measured the length/height of each child. Height-for age and BMI-for-age z-scores were calculated using the ‘zscore06’ STATA command, which uses WHO 2006 growth standards [12]. Stunting was defined as z scores for height-for-age (HAZ) of <-2SD. Wasting was defined as z scores for weight-for-height (WHZ) <-2 SD. Overweight was defined as z scores for WHZ >2 SD and ≤ 3 SD of the median and obese was defined as z scores for WHZ > 3 SD.

### Socioeconomic characteristics

The economic status of households was based on asset indices. Household questionnaires collect information on household assets (such as televisions, refrigerators, and other appliances), dwelling characteristics (materials used for the walls, floor and roof, presence of electricity, water supply and sanitary facilities) and other variables associated with wealth (such as ownership of the house, of land or livestock). A wealth index is constructed using household asset data via principle component analysis, which can be split into quintiles, where the first quintile (Q1) represents the approximately 20% poorest households in the survey sample and the fifth quintile (Q5) represents the richest. Maternal education was self-reported and categorised in four groups: none (E1; no formal education); primary (E2; any primary education, including completed primary education); secondary (E3; any secondary education, including complete secondary) or higher (E4). Urban or rural location were defined by the national census or statistical bureau in each country at the time the survey was conducted.

### Statistical analysis

Regional prevalence estimates and 95% confidence intervals (CIs) for stunting, wasting, overweight (including obesity), and concurrent stunting and overweight including obesity in children <5 years were calculated. The population-level double burden of child malnutrition was defined as the coexistence of overnutrition (overweight including obesity) alongside undernutrition (stunting or wasting). We additionally calculate the individual-level double burden of child malnutrition (i.e. concurrent stunting and overweight including obesity in children included within the study data). Calculation of regional prevalence estimates was completed in two steps. First, we calculated country-specific malnutrition estimates. The DHS used complex sample designs therefore we accounted for stratification and clustering. Second, meta-analysis of country-specific estimates were performed to calculate the regional prevalence estimates and 95% CIs. Variances of the raw proportions were stabilized with a double arcsine transformation and then pooled on the basis of a random-effects model.

We describe and measure socioeconomic and urban-rural inequalities in the population-level double burden of child malnutrition using several approaches. First, we used equiplots (a data visualisation method) to show inequalities, in which each dot represents the prevalence of child malnutrition in a given wealth quintile, maternal education quartile, or urban or rural residence. We also estimated the absolute difference between poorest and richest households, lowest and highest maternal education, and urban and rural residence. Second, we used regression-based measures, slope index of inequality (SII) and relative index of inequality (RII) [13–15], to quantify socioeconomic inequalities in the double burden of child malnutrition at the regional level. A logistic regression model was used to generate SII and RII estimates and 95% CIs. The SII is an absolute measure of inequality that represents the difference in child malnutrition prevalence between the most-advantaged and most-disadvantaged subgroup, while taking into consideration the situation in all other subgroups. Subgroups are weighted according to their population share. If there is no inequality, SII takes the value of zero. Greater absolute values indicate higher levels of inequality. Positive values indicate a prevalence of child malnutrition among the advantaged and negative values indicate a prevalence of child malnutrition among the disadvantaged.

The RII is a relative measure of inequality that represents the ratio of estimated child malnutrition prevalence of the most-advantaged to the most-disadvantaged subgroup, while considering the situation in all other subgroups. Subgroups are weighted according to their population share. If there is no inequality, RII takes the value of one. RII assumes only positive values. The further the value of RII from one, the higher the level of inequality. Values larger than one indicate a greater prevalence of child malnutrition among the advantaged and values smaller than one indicate a greater prevalence of child malnutrition among the disadvantaged.

We used random-effects meta-analysis to average socioeconomic inequality estimates across countries in the East and Southern African region. All of the statistical analyses were performed with the use of STATA version 16.1 (StataCorp). We used the ‘svy’ command to account for complex survey sampling designs and the sampling weights for all countries’ surveys.

## Results

Out of the 17 priority East and Southern African countries, two countries (Mauritius and Seychelles) did not have a DHS dataset, one country’s (Botswana) DHS dataset was restricted, and one country (Madagascar) did not have available anthropometric and child age. In total, 13 out of 17 (77.0%) countries and 75,894 children aged 0–5 years living were eligible for inclusion in our study. Of these children 4.0% (n = 3037) had implausible HAZ and 0.8% (n = 626) had implausible WHZ. Therefore, 72,231 children (95.2%) were included in our final analytical sample.

Table 1 shows the country-level characteristics of participants. The mean age of children was 28.4 months (range: 26.0–29.8 months). The proportion of children with mothers who had no formal education ranged from 1.0% (Lesotho) to 48.0% (Comoros), and the proportion of children who lived in households in the lowest household wealth quintile ranged from 21.6% (Lesotho) to 25.8% (Comoros). The proportion of children living in urban areas ranged from 13.0% (Malawi) to 56.9% (South Africa).

**Table 1:** Characteristics of 13 Demographic Health Surveys in the East and Southern African region.

Country	Year	Children < 5 years (n)	Analytic sample (n)	Response Rate (%)	Mean age (months)	Living in urban area (%)	Lowest household wealth quintile (%)	No maternal education (%)
Comoros	2012	2,882	2,432	84.4	28.6	26.6	25.8	48.0
Eswatini	2006	2,220	2,042	92.0	27.7	18.7	22.0	9.2
Kenya	2015	19,255	18,648	96.8	29.1	34.3	24.2	11.9
Lesotho	2014	1,367	1,303	95.3	27.0	27.3	21.6	1.0
Malawi	2015	5,357	5,116	95.5	29.6	13.1	23.8	13.0
Mozambique	2011	9,772	9,363	95.8	27.8	26.9	23.8	37.7
Namibia	2013	1,945	1,800	92.5	26.0	42.6	25.2	6.9
Rwanda	2014	3,615	3,544	98.0	28.4	16.5	24.8	14.3
South Africa	2016	1,451	1,070	73.7	29.8	56.9	24.1	19.8
Tanzania	2015	9,196	8,940	97.2	28.0	25.9	24.7	21.4
Uganda	2016	4,506	4,382	97.2	28.8	20.4	22.7	11.1
Zambia	2018	9,094	8,694	95.6	28.8	35.0	25.1	10.1
Zimbabwe	2015	5,234	4,897	93.6	29.4	29.6	24.4	1.2

**Fig 1:**
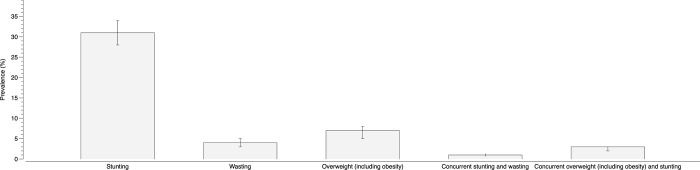


Fig 1 shows the regional prevalence estimates for child malnutrition. Overall, stunting prevalence was 30.5% (95% CI 27.7,33.4), with prevalence ranging from 22.3% (Namibia) to 42.8% (Mozambique) (S1 Table); wasting prevalence was 4.6% (95% CI: 3.7–5.6), with prevalence ranging from 2.3% (Rwanda) to 11.7% (Comoros) (S2 Table); and overweight (including obesity) prevalence was 6.5% (95% CI 5.2,7.9), with prevalence ranging from 3.7% (Tanzania) to 13.5% (South Africa) (S3 Table). The pooled prevalence of concurrent stunting and overweight (including obesity) was 2.6% (95% CI 1.9, 3.5), with the prevalence varying across countries, ranging from 1.2% (Kenya) to 5.2% (Comoros) (S4 Table).

**Fig 2:**
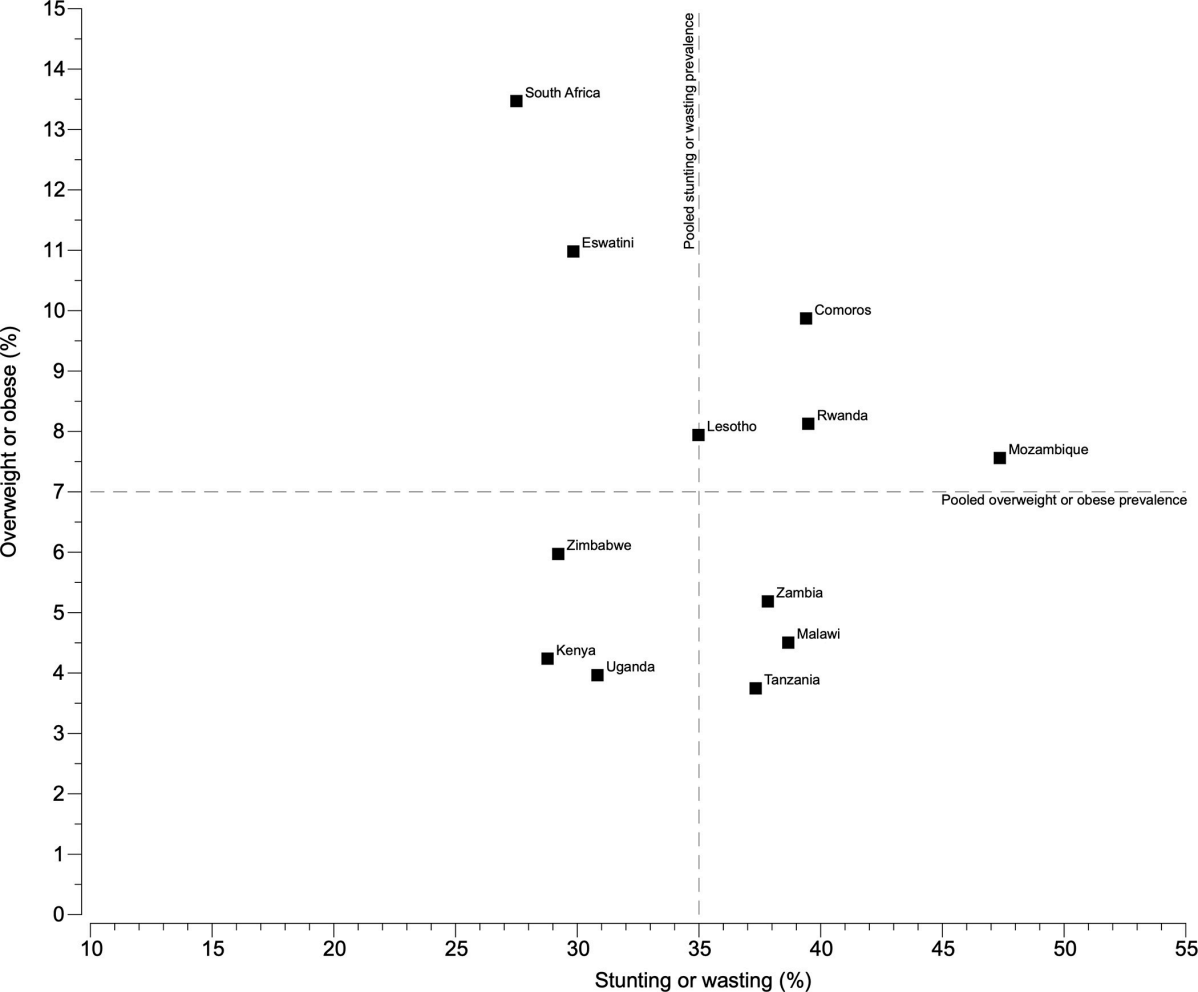


Fig 2 is a visual representation of the double burden of child malnutrition across the region. The country prevalences of overnutrition (overweight including obesity) were plotted by prevalence of undernutrition (stunting or wasting). Each country in the region was represented by one square. The vertical line and the horizontal line separate four quadrants. The vertical line represents the average (or pooled) regional prevalence of undernutrition and the horizontal line represent the average regional prevalence of overnutrition. The plot identifies 23% (3 of 13) of East and Southern African countries (Comoros, Rwanda, and Mozambique) had prevalences of overnutrition and undernutrition greater than the regional average prevalence estimates for overnutrition and undernutrition, respectively.

**Fig 3:**
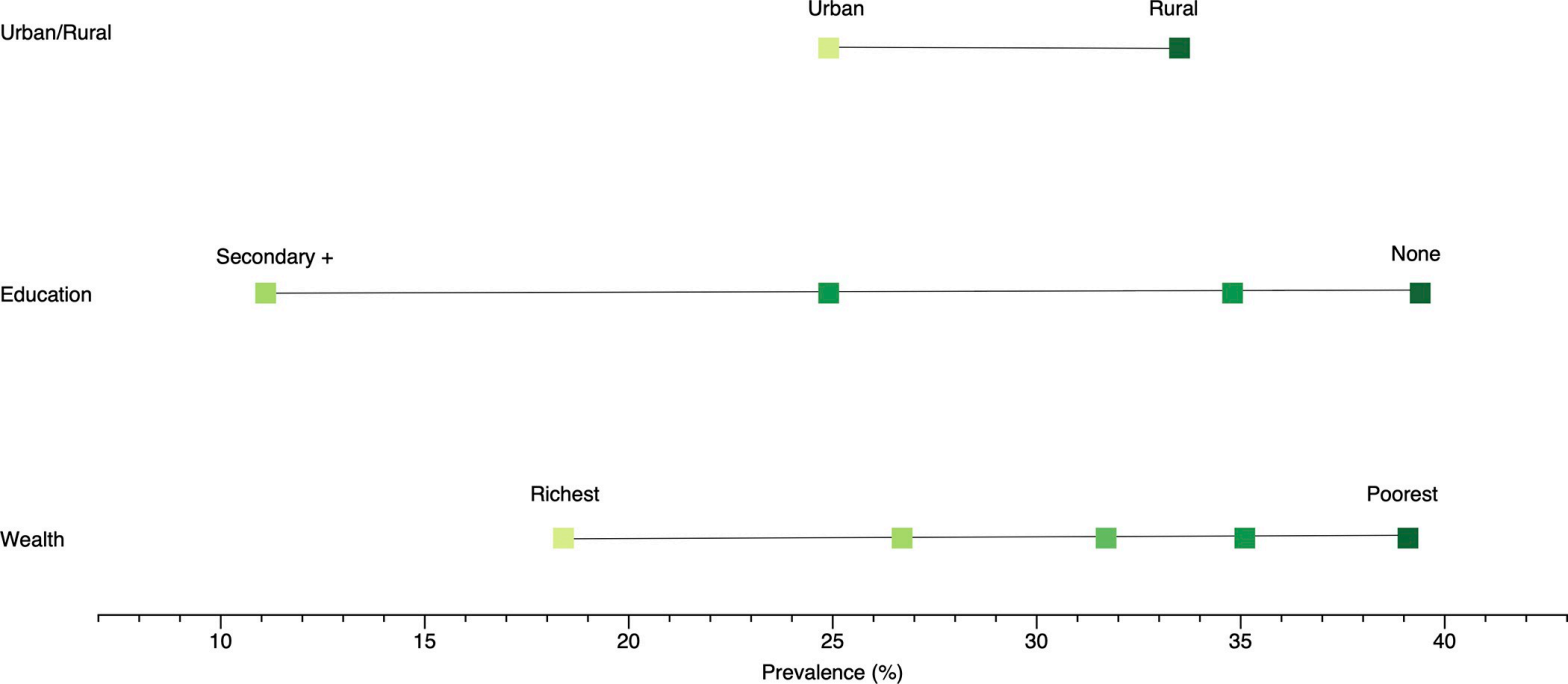


Fig 3 presents equiplots of regional stunting prevalence by wealth quintiles, categories of formal maternal education, and urban-rural residence. Higher stunting prevalence was found among children belonging to the poorest households, mothers with the lowest educational level and rural residence. Lesotho represents an extreme example with the widest gaps in stunting prevalence by wealth (30.5% points; p<0.001) and maternal education (54.1% points; p<0.001) (S6 and S7 Tables). Rwanda showed the widest gap in stunting prevalence by urban-rural residence gap (18.9% points; p<0.001) (S8 Table).

**Fig 4:**
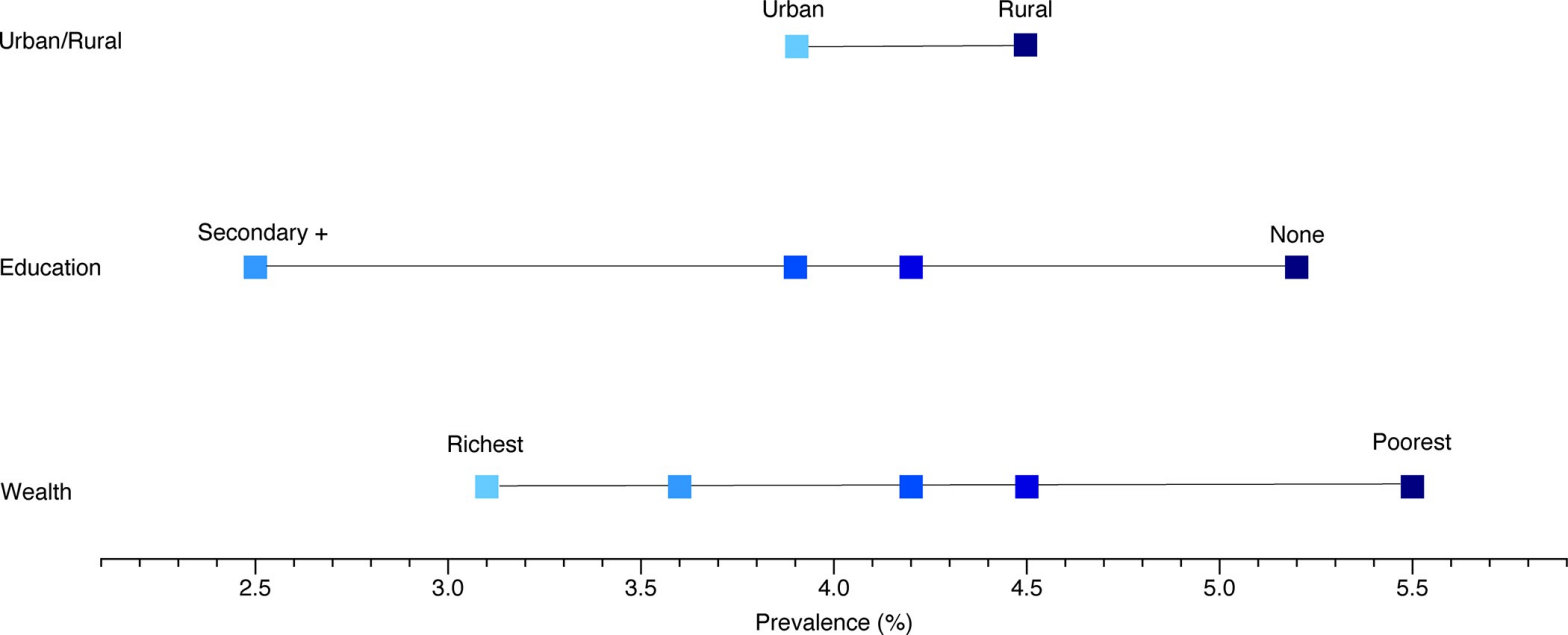


Fig 4 presents equiplots of regional wasting prevalence by wealth quintiles, categories of formal maternal education categories, and urban-rural residence for the region. Higher wasting prevalence was found among children belonging to the poorest households, mothers with the lowest educational level, and rural residence. Mozambique and Namibia represent extreme examples with the widest gaps in wealth (7.1% points; p<0.001) and maternal education gaps (16.2% points; p<0.001) (S9 and S10 Tables). Mozambique showed the widest gap in wasting prevalence by urban-rural residence (3.0%; p<0.001) (S11 Table).

**Fig 5:**
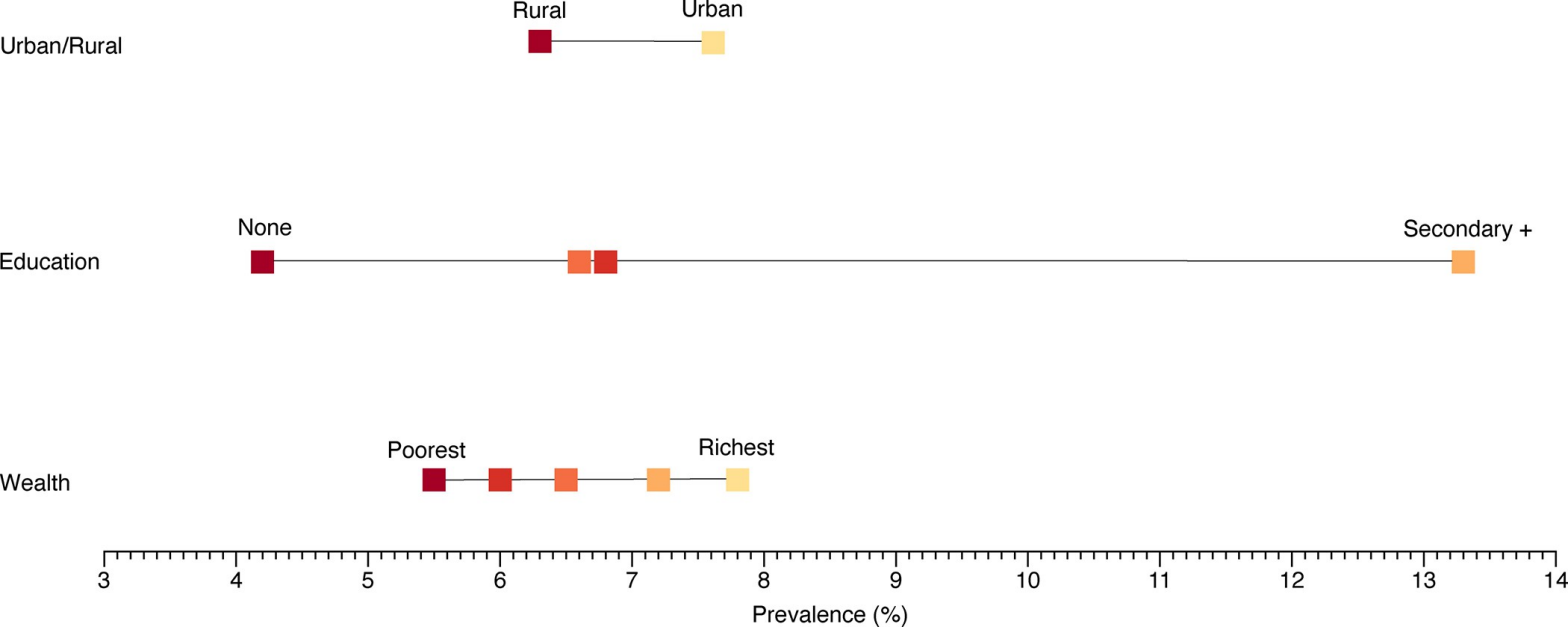


Fig 5 presents equiplots of overweight (including obesity) prevalence by wealth quintiles, categories of formal maternal education categories, and urban-rural residence for the region. Higher overweight (including obesity) prevalence was found among children belonging to the richest households, mothers with the highest educational level and urban residence. Eswatini represents an extreme example, with the widest gaps in overweight (including obesity) prevalence by wealth (6.1% points; p = 0.058), maternal education (12.2% points; p<0.001) and urban-rural residence (6.8% points; p = 0.002) (S12–S14 Tables).

**Fig 6:**
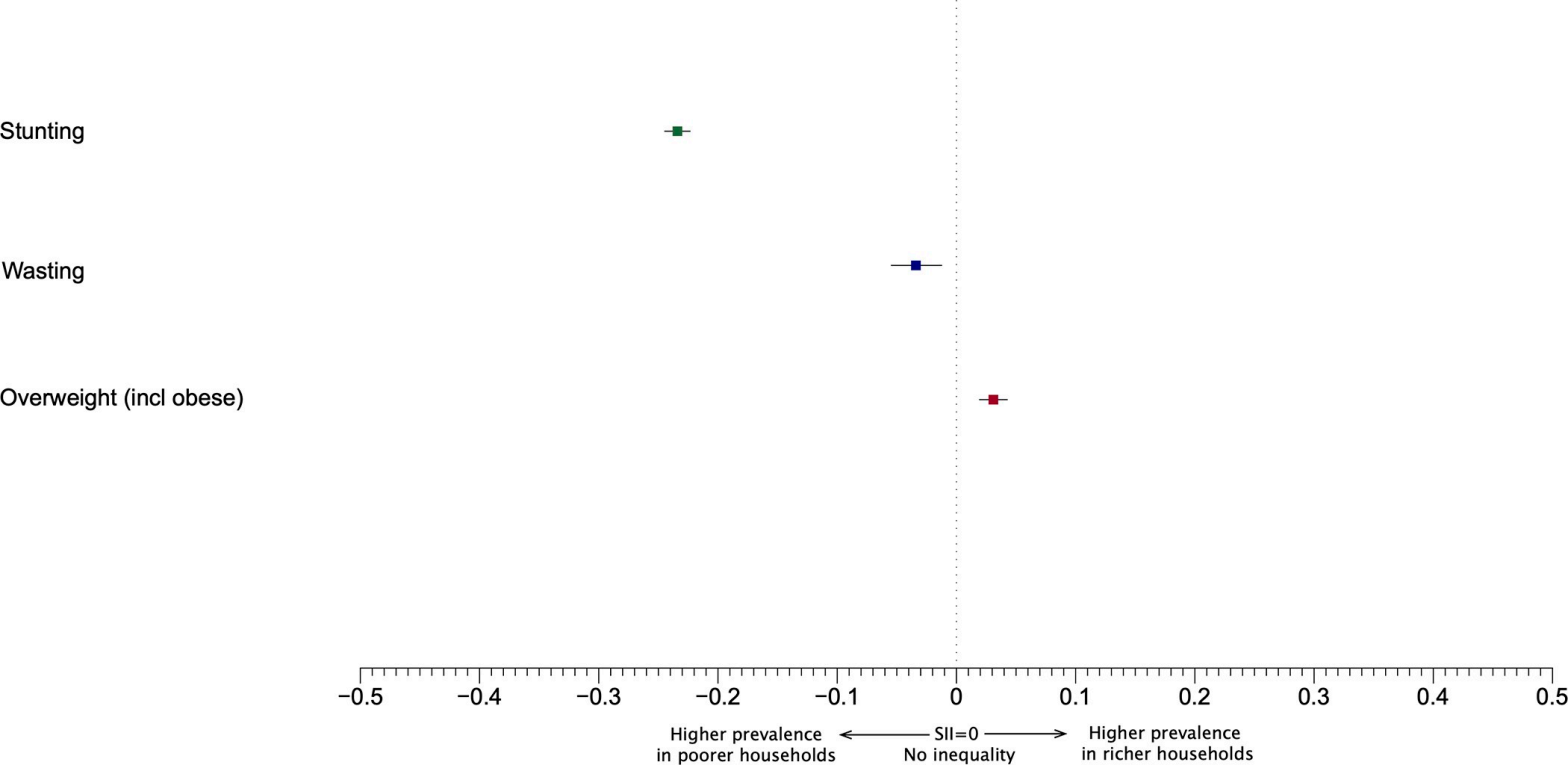


**Fig 7:**
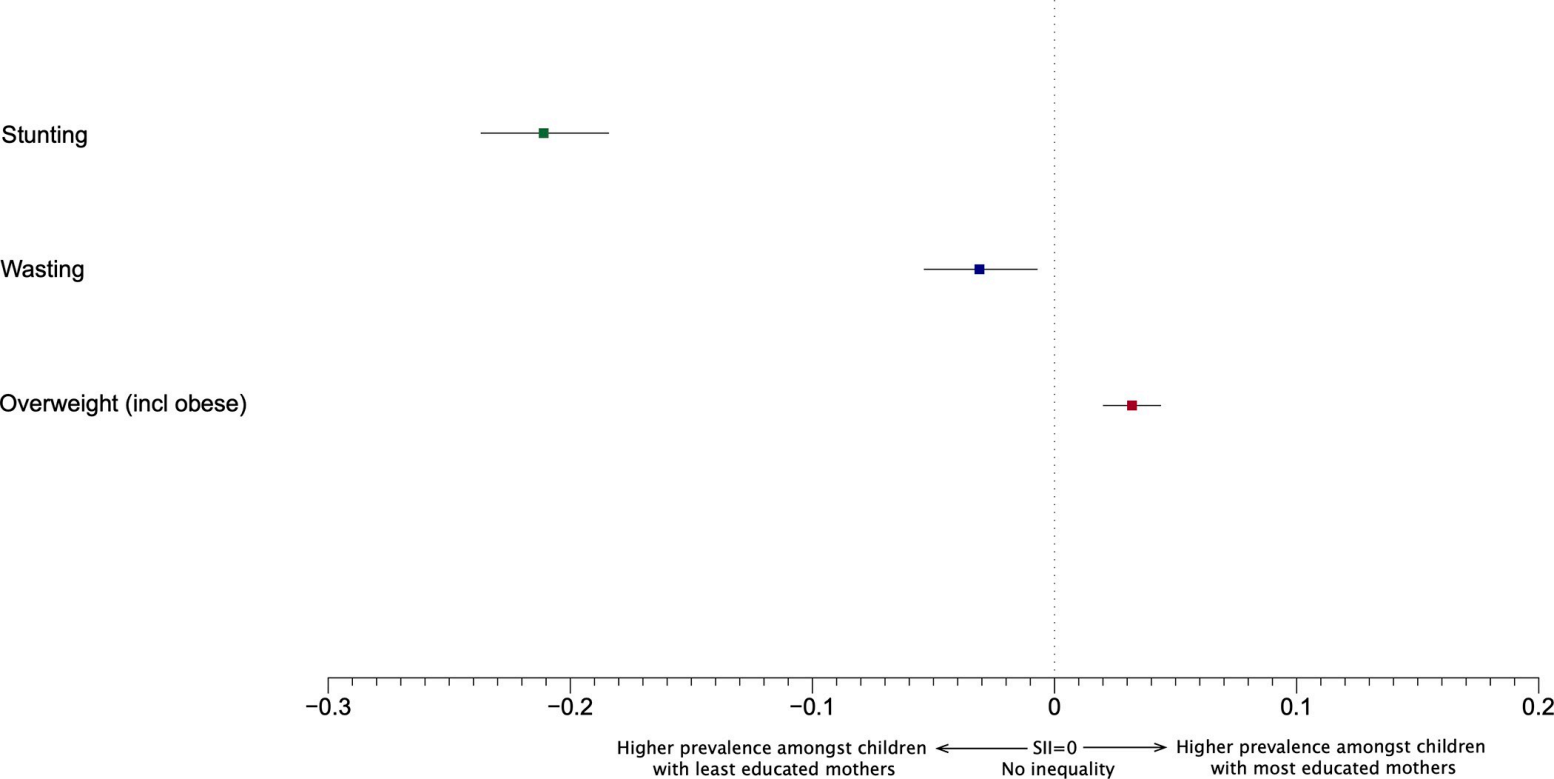


**Fig 8:**
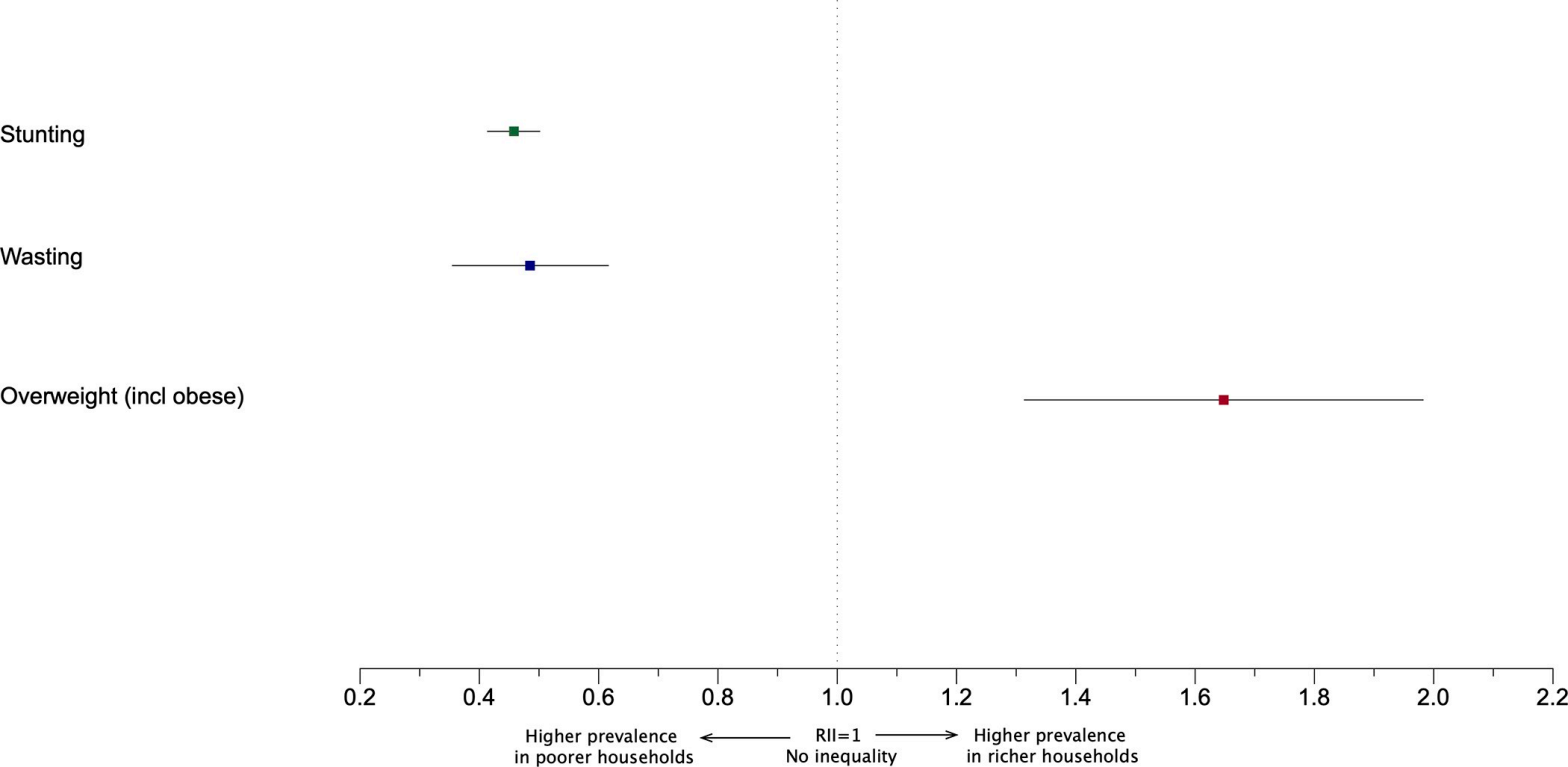


**Fig 9:**
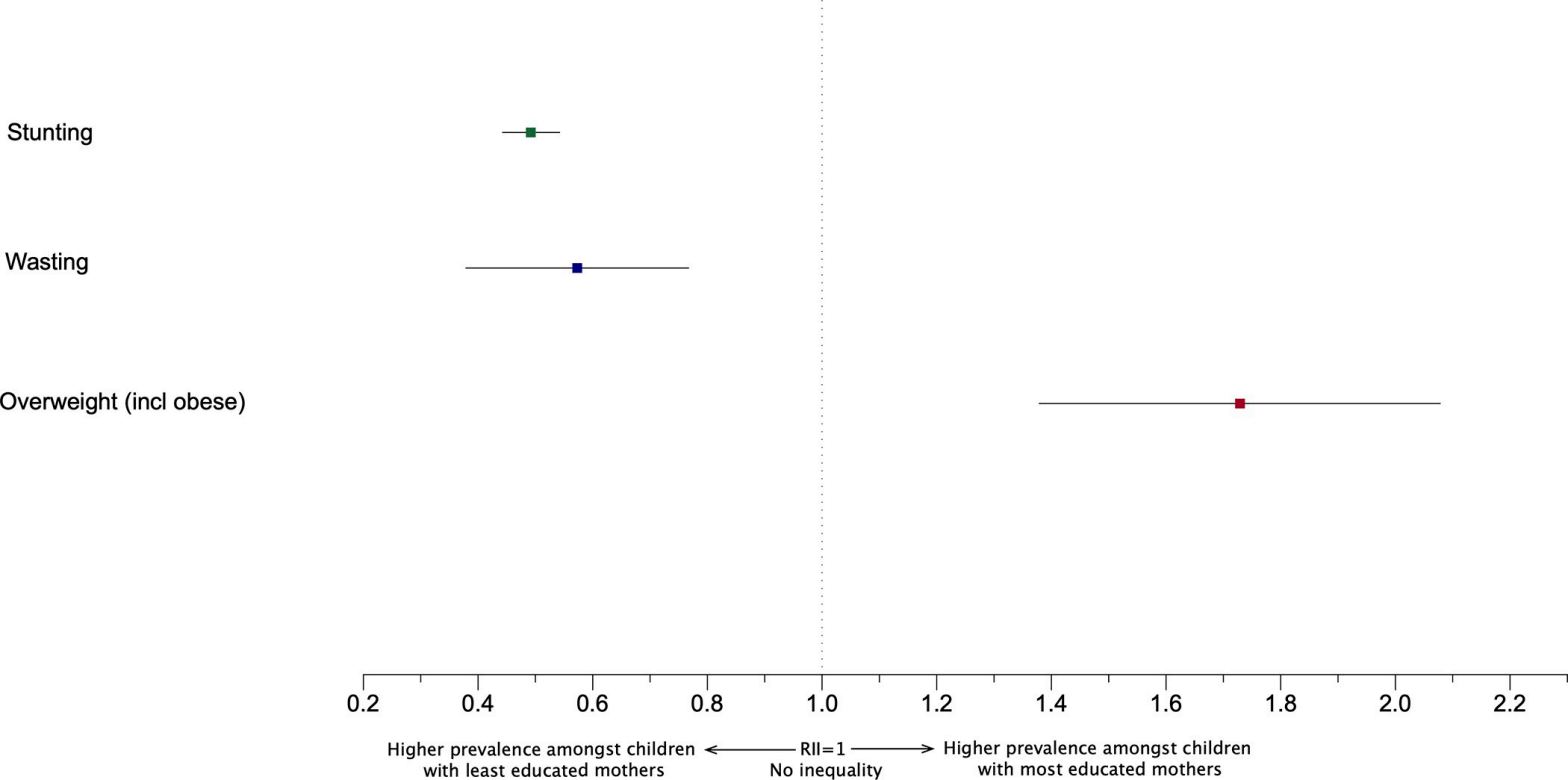


Figs 6–9 show the mean values of stunting, wasting and overweight (including obesity) prevalence respectively, and summary inequality measures. Significant levels of relative and absolute inequalities in the poorest households and least educated mothers were observed for child stunting and wasting. In the absolute scale, the largest wealth inequality in stunting prevalence was in Rwanda (SII = -0.36; 95% CI 0.41,-0.31) and in the relative scale, the largest wealth inequality was in Namibia (RII = 0.36, 95% CI 0.25, 0.47) (S14 Table). The largest maternal education inequality in stunting prevalence was in Rwanda on the absolute scale (SII = -0.33, 95% CI -0.39, -0.26) and South Africa on the relative scale (RII = 0.28, 95% CI 0.15, 0.42) (S16 Table). Kenya had the largest inequalities in child wasting prevalence by household wealth quintile (SII: -0.10, 95% CI -0.12, -0.09; RII: 0.17, 95% CI 0.13, 0.21) and maternal education (SII: -0.13, 95% CI -0.15, -0.11; RII: 0.10 95% CI 0.08, 0.13) (S17 and S18 Tables). In contrast, there were inequalities in the other direction for overweight (including obesity), that is, overweight (including obesity) was concentrated towards the richest wealth quintiles and higher maternal education. In the absolute scale, the largest wealth inequality in overweight (including obese) prevalence was in Comoros (SII: 0.09, 95% CI 0.04, 0.13) and in the relative scale, Kenya (RII: 4.46, 95% CI 3.24, 4.67) (S19 Table). The largest maternal education inequality in overweight (including obese) prevalence on the absolute scale was in Eswatini (SII: 0.11, 95% CI 0.05, 0.16) and Kenya on the relative scale (RII: 4.35, 95% CI 3.09, 5.60) (S20 Table).

## Discussion

To our knowledge, this study is the first to systematically identify the magnitude of socioeconomic and urban-rural inequalities in the population-level double burden of malnutrition among children under five years across the East and Southern African region. We documented socioeconomic inequalities on both on the relative and absolute scales in stunting, wasting and overweight (including obesity), as well as urban-rural inequalities. Pro-poor inequalities in child undernutrition (i.e. undernutrition is more frequent in the children from the poorest households or mothers with the lowest educational level) and pro-rich inequalities in child overnutrition (i.e. overnutrition is more frequent in children from the richest households or mothers with the highest education level) were present in the East and Southern African region.

A study of 55 low-income and middle-income countries (LMICs) between 1992–2018 examining inequalities in double burden of malnutrition found a higher probability of the double burden of malnutrition among richer households in poorer LMICs and poorer households in richer LMICs [16]. The authors defined the double burden of malnutrition at the household level, expressed as a stunted child with an overweight mother. However, there is strong evidence that stunted child/overweight mother pairs represents a statistical artefact and not a distinct entity [17]. Our study assessed socioeconomic inequalities in the double burden of child malnutrition at the population level, which is a common double burden of malnutrition operational definition [4]. A systematic review examining socioeconomic status and overweight or obesity among older children (5–19 years) in Sub-Saharan Africa used the RII to assess socioeconomic inequalities. The school-age children from higher socioeconomic households tended to be overweight and obese [18]. We found similar wealth and education inequalities using both simple and complex absolute and relative measures in the East and Southern African region for children under five years old. In highly developed countries, a higher prevalence of child overweight and obesity children is typically found in lower socioeconomic groups [19]. East and Southern African households of lower socioeconomic status, may have financial constraints that prevent purchasing of high-energy density foods with a consequence that the overweight and obesity prevalence levels are kept to a minimum in these households. Additionally, parent’s socio-cultural beliefs may influence their child’s body shape ideals. In some sub-Saharan African countries, overweight and obesity have historically been considered to be a sign of wealth [18].

The double burden of child malnutrition can occur at different levels including the population-level and individual-level. Our findings indicate in the East and Southern African region, the magnitude of the double burden of child malnutrition at the individual level was markedly smaller than magnitude of the double-burden of child malnutrition at the population-level. The presence of a double burden of malnutrition at a population level, is likely to reflect the region’s ongoing challenges with poverty, food insecurity, infectious diseases, droughts, floods, and conflict as well as the presence of the obesogenic environment driven by globalization and rapid urbanization [5].

Several limitations should be considered when interpreting our findings. The East and Southern African region encompasses 20 countries. Seventeen countries within the region were considered as priority countries by the WHO Intercountry Support Team for East and Southern Africa (H. Bekele 2019, pers. comm., 25 Sept) however only 13 were available in the DHS database. A direct indicator of household income is not collected in the DHS and the asset-based household wealth index was used. While this is an imperfect measure financial resources, it is frequently used and judged to be superior to income in lower income countries [20]. Collection of income or consumption expenditure data in household surveys is challenging. Income can be highly variable from month to month or difficult to accurately measure. Consumption expenditure data can time consuming and expensive to collect [21]. Between country comparisons may be affected by the period of data collection for each national household survey (from 2006 to 2018). Social reform and safety net programmes in the region have increased over this study period and may have reduced inequalities over time [22]. We adopted a single level modelling approach our analyses and did not consider the context in which processes may occur. Factors such as climate variability, internal conflict, governance and gross domestic product (GDP) should be taken into consideration when formulating policy on the double burden of malnutrition in the East and Southern African region.

Despite these limitations, our study has several strengths. Our analysis of socioeconomic and urban-rural inequalities in child malnutrition in the East and Southern African region are based on a relatively large and diverse sample of children from 13 nationally representative household surveys with uniformity of information based on the same questions and procedures in all countries. We used both simple and complex measures to show inequality. Simple measures only compare two subgroups. For example, we used equiplots to compare the richest and poorest. However, simple measures cannot describe how all groups change. We also used complex regression-based measures (including SII and RII) which indicated socioeconomic inequality across the whole population between the most and the least disadvantaged. We reported socioeconomic inequalities on both scales because conclusions can be skewed when only one or the other is used [14]. Both these measures consider the size of the population across wealth and education groups.

Addressing socioeconomic and urban-rural inequalities in the distribution of the double burden of malnutrition among children in the East and Southern African region requires strategies that address the reasons certain subgroups became more exposed to these nutrition problems, while also avoiding strategies that solve one nutrition problem while worsening another one [7]. For example, to avoid inadvertently promoting overweight and obesity by endorsing the high consumption of energy-dense but not necessarily micronutrient-rich foods, beneficiaries of supplementary food programmes should not be selected solely on socioeconomic status, but on nutritional assessment and monitored for changing needs and true effectiveness [23,24]. Programs and policies to address the double burden of child malnutrition in the East and Southern African region, will require robust nutrition surveillance systems to ensure they have equitable reach and quality. Traditionally, child nutrition surveillance systems relied on household or other population-based surveys. However, these are typically high cost, low frequency, and are unable to provide district-level estimates [25]. To provide near-real time child nutrition data linked to both large and small geographic areas, further attention has to be given to using nutrition data from growth monitoring programmes in primary care clinics.

The aftermath of the coronavirus disease 2019 (COVID-19) pandemic, surging oil and food prices are likely to be a time of worsening socioeconomic conditions and reduced public spending [26,27]. Policymakers seeking to address the double burden of malnutrition need to make careful decisions regarding the targeting of limited resources, given that both pro-poor inequalities in child undernutrition and pro-rich inequalities in child overweight (including obesity) are present in the East and Southern African region.

## Supporting information

S1 Table Country-specific prevalence estimates for stunting among children aged < 5 years in 13 East and Southern African countries from the DHS. (DOCX)

S2 Table Country-specific prevalence estimates for wasting among children under five in 13 East and Southern African countries from the DHS. (DOCX)

S3 Table Country-specific prevalence estimates for overweight (including obesity) among children under five in 13 East and Southern African countries from the DHS. (DOCX)

S4 Table Country-specific prevalence estimates for concurrent overweight (including obesity) and stunting among children under five in 13 East and Southern African countries from the DHS. (DOCX)

S5 Table Country-specific prevalence estimates for concurrent wasting and stunting among children aged under five in 13 East and Southern African countries from the DHS. (DOCX)

S6 Table Wealth quintile differentials in child stunting by country and year. (DOCX)

S7 Table Maternal education differentials in child stunting by country and year. (DOCX)

S8 Table Residence differentials in child stunting by country and year. (DOCX)

S9 Table Wealth index differentials in child wasting by country and year. (DOCX)

S10 Table Maternal education differentials in child wasting by country and year. (DOCX)

S11 Table Residence differentials in child wasting by country and year. (DOCX)

S12 Table Wealth index differentials of child overweight (including obesity) by country and year. (DOCX)

S13 Table Maternal education differentials of child overweight (including obesity) by country and year. (DOCX)

S14 Table Residence differentials of child overweight (including obesity) by country and year. (DOCX)

S15 Table Country-specific household wealth gradient of stunting among children under five—slope index of inequality (SII) and relative index of inequality (RII) on magnitude of inequality in stunting. (DOCX)

S16 Table Country-specific maternal education gradient of stunting among children under five—slope index of inequality (SII) and relative index of inequality (RII) on magnitude of inequality in stunting. (DOCX)

S17 Table Country-specific household wealth gradient of wasting among children under five—slope index of inequality (SII) and relative index of inequality (RII) on magnitude of inequality in wasting. (DOCX)

S18 Table Country-specific maternal education gradient of wasting among children under five—slope index of inequality (SII) and relative index of inequality (RII) on magnitude of inequality in wasting. (DOCX)

S19 Table Country-specific household wealth gradient of overweight (including obesity) among children under five—slope index of inequality (SII) and relative index of inequality (RII) on magnitude of inequality in overweight (including obesity). (DOCX)

S20 Table Country-specific maternal education gradient of overweight (including obesity) among children under five—slope index of inequality (SII) and relative index of inequality (RII) on magnitude of inequality in overweight (including obesity). (DOCX)
