# Supplementary material for: Socioeconomic and urban-rural inequalities in the population-level double burden of child malnutrition in the East and Southern African Region
Source: PLOS Glob Public Health. 2023 Apr 25;3(4):e0000397. doi: 10.1371/journal.pgph.0000397 (PMC10128925; doi:10.1371/journal.pgph.0000397)
Supplement: S1 Table — (DOCX) [file pgph.0000397.s001.docx]

**S1 Table**. Country-specific prevalence estimates for stunting among children aged < 5 years in 13 East and Southern African countries from the DHS

|  | N | Stunting prevalence  95% CI |
| --- | --- | --- |
| Comoros 2012 | 681 | 30.0 (27.6-32.5) |
| Eswatini 2006 | 552 | 27.7 (25.6-30.) |
| Kenya 2014 | 5054 | 25.9 (24.9-26.9) |
| Lesotho 2014 | 450 | 32.5 (29.5-35.8 |
| Malawi 2015-16 | 1807 | 36.7 (35.0-35.0) |
| Mozambique 2011 | 3679 | 42.8 (41.2-44.5) |
| Namibia 2013 | 401 | 21.1 (18.8-23.5) |
| Rwanda 2014-15 | 1332 | 37.9 (36.0-39.7) |
| South Africa 2016 | 264 | 25.4 (22.0-29.2) |
| Tanzania 2015-16 | 2994 | 34.1 (32.6-35.6 |
| Uganda 2016 | 1242 | 28.1 (26.4-29.9) |
| Zambia 2018 | 3018 | 34.4 (33.2-35.7) |
| Zimbabwe 2015 | 1248 | 26.2 (24.6-27.9) |
