# Supplementary material for: Socioeconomic and urban-rural inequalities in the population-level double burden of child malnutrition in the East and Southern African Region
Source: PLOS Glob Public Health. 2023 Apr 25;3(4):e0000397. doi: 10.1371/journal.pgph.0000397 (PMC10128925; doi:10.1371/journal.pgph.0000397)
Supplement: S2 Table — (DOCX) [file pgph.0000397.s002.docx]

**S2 Table**. Country-specific prevalence estimates for wasting among children under five in 13 East and Southern African countries from the DHS

|  | N | Wasting prevalence  95% CI |
| --- | --- | --- |
| Comoros 2012 | 298 | 11.7 (10.2,13.4) |
| Eswatini 2006 | 53 | 2.6 (2.0,3.5) |
| Kenya 2014 | 1058 | 4.2 (3.8,4.7) |
| Lesotho 2014 | 49 | 3.3 (2.4,4.5) |
| Malawi 2015-16 | 168 | 2.9 (2.4,3.5) |
| Mozambique 2011 | 517 | 6.3 (5.7,7.0) |
| Namibia 2013 | 153 | 8.4 (7.0-10.2) |
| Rwanda 2014-15 | 81 | 2.3 (1.9,2.9) |
| South Africa 2016 | 31 | 2.6 (1.6,4.1) |
| Tanzania 2015-16 | 449 | 4.9 (4.3,5.4) |
| Uganda 2016 | 170 | 3.8 (3.2,4.5) |
| Zambia 2018 | 370 | 4.5 (3.9,5.2) |
| Zimbabwe 2015 | 182 | 3.8 (3.2,4.4) |
