# Supplementary material for: Socioeconomic and urban-rural inequalities in the population-level double burden of child malnutrition in the East and Southern African Region
Source: PLOS Glob Public Health. 2023 Apr 25;3(4):e0000397. doi: 10.1371/journal.pgph.0000397 (PMC10128925; doi:10.1371/journal.pgph.0000397)
Supplement: S3 Table — (DOCX) [file pgph.0000397.s003.docx]

**S3 Table**. Country-specific prevalence estimates for overweight (including obesity) among children under five in 13 East and Southern African countries from the DHS

|  | N | Overweight (including obesity) prevalence  95% CI |
| --- | --- | --- |
| Comoros 2012 | 233 | 9.9 (8.4,11.6) |
| Eswatini 2006 | 225 | 11.0 (9.6,12.6) |
| Kenya 2014 | 676 | 4.2(3.8,4.7) |
| Lesotho 2014 | 107 | 7.9(6.5,9.7) |
| Malawi 2015-16 | 219 | 4.5(3.8,5.3) |
| Mozambique 2011 | 741 | 7.6(6.8,8.3) |
| Namibia 2013 | 80 | 4.4 (3.5-5.7) |
| Rwanda 2014-15 | 288 | 8.1(7.2,9.1) |
| South Africa 2016 | 138 | 13.5 (10.9,16.5) |
| Tanzania 2015-16 | 319 | 3.7(3.3,4.3) |
| Uganda 2016 | 173 | 4.0(3.3,4.7) |
| Zambia 2018 | 444 | 5.2(4.6,5.9) |
| Zimbabwe 2015 | 307 | 6.0(5.2,6.8) |
