# Supplementary material for: Socioeconomic and urban-rural inequalities in the population-level double burden of child malnutrition in the East and Southern African Region
Source: PLOS Glob Public Health. 2023 Apr 25;3(4):e0000397. doi: 10.1371/journal.pgph.0000397 (PMC10128925; doi:10.1371/journal.pgph.0000397)
Supplement: S4 Table — (DOCX) [file pgph.0000397.s004.docx]

**S4 Table**. Country-specific prevalence estimates for concurrent overweight (including obesity) and stunting among children under five in 13 East and Southern African countries from the DHS

|  | N | Concurrent overweight (including obesity) and stunting prevalence  95% CI |
| --- | --- | --- |
| Comoros (2012) | 125 | 5.2(4.1,6.7) |
| Eswatini (2006) | 75 | 3.8(3.0,4.8) |
| Kenya (2014) | 207 | 1.2(1.0,1.5) |
| Lesotho (2014) | 43 | 2.8(2.0,4.0) |
| Malawi (2015-16) | 101 | 2.1(1.6,2.8) |
| Mozambique (2011) | 427 | 4.8(4.1,5.5) |
| Namibia (2013) | 31 | 1.5 (1.1, 2.2) |
| Rwanda (2014-15) | 110 | 3.1(2.5,3.7) |
| South Africa (2016) | 43 | 4.3(2.9,6.2) |
| Tanzania (2015-16) | 150 | 1.6(1.3,1.9) |
| Uganda (2016) | 56 | 1.2(0.9,1.6) |
| Zambia (2018) | 215 | 2.5(2.0,3.0) |
| Zimbabwe (2015) | 114 | 2.2(1.7,2.7) |
