# Supplementary material for: Socioeconomic and urban-rural inequalities in the population-level double burden of child malnutrition in the East and Southern African Region
Source: PLOS Glob Public Health. 2023 Apr 25;3(4):e0000397. doi: 10.1371/journal.pgph.0000397 (PMC10128925; doi:10.1371/journal.pgph.0000397)
Supplement: S5 Table — (DOCX) [file pgph.0000397.s005.docx]

**S5 Table**. Country-specific prevalence estimates for concurrent wasting and stunting among children aged under five in 13 East and Southern African countries from the DHS

|  | N | Concurrent wasting and stunting prevalence  95% CI |
| --- | --- | --- |
| Comoros (2012) | 50 | 2.3(1.6,3.1) |
| Eswatini (2006) | 10 | 0.5(0.3,1.0) |
| Kenya (2014) | 322 | 1.3(1.1,1.5) |
| Lesotho (2014) | 14 | 0.9(0.5,1.7) |
| Malawi (2015-16) | 50 | 0.9(0.7,1.3) |
| Mozambique (2011) | 141 | 1.8(1.5,2.1) |
| Rwanda (2014-15) | 24 | 0.7(0.5,1.1) |
| South Africa (2016) | 4 | 0.5(0.1,2.0) |
| Tanzania (2015-16) | 145 | 1.6(1.3,2.0) |
| Uganda (2016) | 50 | 1.1(0.8,1.5) |
| Zambia (2018) | 84 | 1.1(0.8,1.4) |
| Zimbabwe (2015) | 34 | 0.8(0.5,1.1) |
