# Supplementary material for: Socioeconomic and urban-rural inequalities in the population-level double burden of child malnutrition in the East and Southern African Region
Source: PLOS Glob Public Health. 2023 Apr 25;3(4):e0000397. doi: 10.1371/journal.pgph.0000397 (PMC10128925; doi:10.1371/journal.pgph.0000397)
Supplement: S6 Table — (DOCX) [file pgph.0000397.s006.docx]

**S6 Table.** Wealth quintile differentials in child stunting by country and year

|  |  | Wealth index | | | | | | |
| --- | --- | --- | --- | --- | --- | --- | --- | --- |
| **Country and survey year** | **Sample size** | **Q1**  **(95% CI)** | **Q2**  **(95% CI)** | **Q3**  **(95% CI)** | **Q4**  **(95% CI)** | **Q5**  **(95% CI)** | **Gap**  **(% points)** | **p-value**  **(Q1-Q5)** |
| Comoros 2012 | 2,432 | 37.4 (33.1-41.9) | 32.3 (27.5-37.5) | 25.8 (21.0-31.3) | 27.0 (21.5-33.4) | 22.8 (16.7-30.4) | 14.6 | 0.0024 |
| Eswatini 2006 | 2,042 | 37.0 (32.3-41.9) | 31.2 (26.5-36.2) | 25.3 (20.9-30.3) | 24.3 (19.8-29.5) | 17.2 (13.0-22.4) | 19.8 | <0.001 |
| Kenya 2014 | 18,648 | 35.6 (33.8-37.5) | 30.4 (28.5-32.4) | 25.4 (23.3-27.6) | 21.0 (19.0-23.3) | 13.5 (11.5-15.7) | 22.1 | <0.001 |
| Lesotho 2014 | 1,303 | 44.2 (37.7-51.0) | 36.6 (30.4-43.1) | 36.9 (29.7-44.7) | 26.8 (19.0-36.2) | 13.7 (8.5-21.2) | 30.5 | <0.001 |
| Malawi 2015-16 | 5,116 | 45.0 (41.7-48.5) | 40.8 (37.4-44.3) | 36.7 (33.2-40.4) | 31.9 (28.1-36.0) | 23.3 (19.5-27.6) | 21.7 | <0.001 |
| Mozambique 2011 | 9,363 | 50.8 (47.8-53.7) | 47.9 (44.7-51.1) | 46.1 (42.6-49.5) | 38.1 (34.9-41.3) | 24.2 (21.8-26.9) | 26.6 | <0.001 |
| Namibia 2013 | 1,800 | 28.7 (23.9-34.0) | 24.1 (19.4-29.5) | 20.9 (16.7-25.7) | 15.5 (11.8-20.2) | 9.7 (5.4-16.7) | 19.0 | <0.001 |
| Rwanda 2014-15 | 3,544 | 48.6 (45.2-52.0) | 45.8 (42.1-49.6) | 37.9 (34.0-41.9) | 28.9 (25.1-32.9) | 20.6 (17.3-24.3) | 28.0 | <0.001 |
| South Africa 2016 | 1,070 | 35.9 (29.1-43.2) | 27.6 (20.4-36.2) | 23.1 (17.1-30.4) | 21. (14.8-29.4) | 10.0 (4.6-20.0) | 25.9 | <0.001 |
| Tanzania 2015-16 | 8,940 | 39.4 (36.4-42.5) | 38.8 (35.9-41.7) | 38.7 (35.7-41.8) | 29.3 (26.8-31.9) | 19.2 (16.6-22.1) | 20.2 | <0.001 |
| Uganda 2016 | 4,382 | 32.3 (29.3-35.5) | 32.3 (28.4-36.5) | 31.4 (27.9-35.2) | 26.6 (22.7-30.8) | 16.8 (13.8-20.3) | 15.5 | <0.001 |
| Zambia 2018 | 8,694 | 39.9 (37.4-42.4) | 36.4 (34.3-38.6) | 32.6 (30.1-35.3) | 34.9 (32.4-37.5) | 23.9 (20.9-27.3) | 16.0 | <0.001 |
| Zimbabwe 2015 | 4,897 | 32.3 (29.0-35.8) | 27.5 (24.2-31.1) | 25.7 (21.9-29.9) | 26.6 (23.2-30.3) | 15.2 (12.6-18.3) | 17.1 | <0.001 |

Q1, poorest quintile; Q2, poorer quintile; Q3, middle quintile; Q4, richer quintile; Q5, richest quintile.
