# Supplementary material for: Socioeconomic and urban-rural inequalities in the population-level double burden of child malnutrition in the East and Southern African Region
Source: PLOS Glob Public Health. 2023 Apr 25;3(4):e0000397. doi: 10.1371/journal.pgph.0000397 (PMC10128925; doi:10.1371/journal.pgph.0000397)
Supplement: S7 Table — (DOCX) [file pgph.0000397.s007.docx]

**S7 Table.** Maternal education differentials in child stunting by country and year

|  |  | Maternal Education level | | | | | |
| --- | --- | --- | --- | --- | --- | --- | --- |
| **Country and survey year** | **Sample size** | **E1**  **(95% CI)** | **E2**  **(95% CI)** | **E3**  **(95% CI)** | **E4**  **(95% CI)** | **Gap**  **(% points)** | **p-value**  **(E1-E4)** |
| Comoros 2012 | 2,422 | 33.1 (29.6-36.9) | 33.2 (28.4-38.4) | 24.7 (21.9-31.7) | 12.6 (6.2-23.7) | 18.7 | 0.0005 |
| Eswatini 2006 | 2,042 | 38.8 (31.0-47.2) | 33.3 (29.9-36.9) | 23.5 (20.7-26.5) | 10.7 (6.2-17.7) | 28.1 | <0.001 |
| Kenya 2014 | 18,648 | 30.5 (27.7-33.4) | 29.7 (28.4-31.1) | 19.0 (17.2-20.8) | 12.5 (10.3-15.1) | 18.0 | <0.001 |
| Lesotho 2014 | 1,303 | 71.7 (52.0-85.6) | 37.5 (33.5-41.7) | 29.1 (24.8-33.7) | 17.6 (9.3-30.7) | 54.1 | <0.001 |
| Malawi 2015-16 | 5,116 | 43.5 (38.9-48.1) | 38.0 (35.8-40.1) | 29.9 (26.7-33.4) | 13.0 (4.7-31.0) | 30.5 | <0.001 |
| Mozambique 2011 | 9,363 | 47.1 (44.9-49.3) | 43.1 (41.0-45.3) | 27.7 (24.4-31.3) | 9.8 (4.1-21.6) | 37.3 | <0.001 |
| Namibia 2013 | 1,800 | 32.7 (25.0-41.5) | 27.8 (23.0-33.2) | 18.4 (15.8-21.3) | 7.3 (2.2-21.6) | 25.4 | <0.001 |
| Rwanda 2014-15 | 3,544 | 47.3 (42.8-51.9) | 39.3 (37.1-41.5) | 22.3 (18.3-26.9) | 5.2 (2.1-12.4) | 42.1 | <0.001 |
| South Africa 2016* | 1,070 | 46.7 (21.9-73.3) | 35.8 (26.0-47.0) | 26.2 (22.5-30.2) | 5.0 (2.0-11.8) | 41.7 | <0.001 |
| Tanzania 2015-16 | 8,940 | 39.3 (36.5-42.1) | 34.7 (33.0-36.6) | 24.1 (21.1-27.3) | 8.5 (3.5-19.0) | 30.8 | <0.001 |
| Uganda 2016 | 4,382 | 38.0 (33.0-43.3) | 30.0 (27.9-32.2) | 23.0 (19.7-26.8) | 9.4 (5.8-15.0) | 28.6 | <0.001 |
| Zambia 2018 | 8,694 | 37.7 (33.5-42.1) | 37.4 (35.8-38.9) | 31.1 (29.0-33.4) | 15.1 (11.0-20.3) | 22.6 | <0.001 |
| Zimbabwe 2015 | 4,897 | 44.6 (31.0-59.0) | 31.2 (28.3-34.2) | 24.7 (22.8-26.7) | 8.8 (5.7-13.4) | 35.8 | <0.001 |

E1, no education; E2, primary education; E3, secondary education; E4, higher education
