# Supplementary material for: Socioeconomic and urban-rural inequalities in the population-level double burden of child malnutrition in the East and Southern African Region
Source: PLOS Glob Public Health. 2023 Apr 25;3(4):e0000397. doi: 10.1371/journal.pgph.0000397 (PMC10128925; doi:10.1371/journal.pgph.0000397)
Supplement: S8 Table — (DOCX) [file pgph.0000397.s008.docx]

**S8 Table.** Residence differentials in child stunting by country and year.

|  |  | Area of residence | | | |
| --- | --- | --- | --- | --- | --- |
| **Country and survey year** | **Sample size** | **Rural**  **(95% CI)** | **Urban**  **(95% CI)** | **Gap**  **(% points)** | **p-value**  **(rural-urban)** |
| Comoros 2012 | 2,432 | 31.6 (28.8-34.7) | 25.4 (21.6-39.6) | 6.2 | 0.0165 |
| Eswatini 2006 | 2,042 | 28.7 (26.3-31.2) | 23.4 (18.9-28.6) | 5.3 | 0.065 |
| Kenya 2014 | 18,648 | 29.1 (27.9-30.3) | 19.7 (17.9-21.7) | 9.4 | <0.001 |
| Lesotho 2014 | 1,303 | 34.3 (31.0-37.7) | 27.9 (21.3-35.7) | 6.4 | 0.136 |
| Malawi 2015-16 | 5,116 | 38.5 (36.8-40.3) | 24.8 (20.2-29.9) | 13.7 | <0.001 |
| Mozambique 2011 | 9,363 | 45.5 (43.6-47.4) | 35.6 (32.2-39.1) | 9.9 | <0.001 |
| Namibia 2013 | 1,800 | 24.1 (21.1-27.3) | 17.0 (13.6-21.1) | 7.1 | 0.006 |
| Rwanda 2010 | 4,084 | 46.3 (44.2-48.3) | 27.4 (22.5-32.8) | 18.9 | <0.001 |
| South Africa 2016 | 1,070 | 28.1 (24.0-32.6) | 23.4 (18.4-29.3) | 4.7 | 0.193 |
| Tanzania 2015-16 | 8,940 | 37.2 (35.5-39.0) | 25.1 (22.0-28.4) | 12.1 | <0.001 |
| Uganda 2016 | 4,382 | 29.6 (27.7-31.6) | 22.4 (19.1-26.2) | 7.2 | 0.001 |
| Zambia 2018 | 8,694 | 35.7 (34.2-37.2) | 32.0 (29.8-34.2) | 3.7 | 0.006 |
| Zimbabwe 2015 | 4,897 | 28.0 (26.1-30.1) | 22.0 (18.9-25.4) | 6.0 | 0.003 |
