# Supplementary material for: Socioeconomic and urban-rural inequalities in the population-level double burden of child malnutrition in the East and Southern African Region
Source: PLOS Glob Public Health. 2023 Apr 25;3(4):e0000397. doi: 10.1371/journal.pgph.0000397 (PMC10128925; doi:10.1371/journal.pgph.0000397)
Supplement: S9 Table — (DOCX) [file pgph.0000397.s009.docx]

**S9 Table.** Wealth index differentials in child wasting by country and year

|  |  | Wealth index | | | | | | |
| --- | --- | --- | --- | --- | --- | --- | --- | --- |
| **Country and survey year** | **Sample size** | **Q1**  **(95% CI)** | **Q2**  **(95% CI)** | **Q3**  **(95% CI)** | **Q4**  **(95% CI)** | **Q5**  **(95% CI)** | **Gap**  **(% points)** | **p-value**  **(Q1-Q5)** |
| Comoros 2012 | 2,432 | 13.3 (10.4-16.8) | 11.6 (8.6-15.4) | 11.8 (8.5-16.2) | 10.3 (7.3-14.3) | 10.7 (7.0-16.0) | 2.6 | 0.787 |
| Eswatini 2006 | 2,042 | 3.3 (2.0-5.4) | 2.7 (1.5-4.7) | 3.6 (1.9-6.8) | 2.0 (1.0-4.0) | 1.1 (0.4-3.1) | 2.2 | 0.260 |
| Kenya 2014 | 18,648 | 7.6 (6.3-9.0) | 3.3 (2.7-4.1) | 3.8 (3.0-4.9) | 2.6 (2.0-3.5) | 2.7 (2.0-3.6) | 4.9 | <0.001 |
| Lesotho 2014 | 1,303 | 5.8 (3.6-9.2) | 4.8 (2.7-8.5) | 2.8 (1.3-5.8) | 1.8 (0.7-4.7) | 0.7 (0.1-3.8) | 5.1 | 0.015 |
| Malawi 2015-16 | 5,116 | 3.1 (2.1-4.4) | 2.6 (1.8-3.7) | 3.3 (2.2-5.0) | 2.3 (1.5-3.6) | 3.3 (2.0-5.3) | 0.2 | 0.701 |
| Mozambique 2011 | 9,363 | 10.3 (8.6-12.1) | 6.3 (5.0-7.8) | 5.7 (4.7-7.1) | 4.5 (3.3-6.1) | 3.2 (2.3-4.4) | 7.1 | <0.001 |
| Namibia 2013 | 1,800 | 10.6 (8.0-13.9) | 8.1 (5.6-11.6) | 10.6 (7.1-15.6) | 6.2 (3.8-10.1) | 5.2 (2.5-10.4) | 5.4 | 0.121 |
| Rwanda 2014-15 | 3,544 | 2.5 (1.6-3.8) | 2.6 (1.6-4.0) | 2.9 (1.9-4.4) | 1.6 (0.8-3.1) | 1.8 (1.0-3.2) | 0.7 | 0.511 |
| South Africa 2016 | 1,070 | 1.4 (0.5-3.4) | 6.0 (3.0-11.9) | 2.0 (1.0-4.1) | 1.9 (0.7-5.1) | 0.2 (0.0-1.3) | 1.2 | 0.001 |
| Tanzania 2015-16 | 8,940 | 5.3 (4.2-6.6) | 5.0 (4.0-6.3) | 5.8 (4.4-7.6) | 4.3 (3.3-5.5) | 3.6 (2.4-5.2) | 1.7 | 0.181 |
| Uganda 2016 | 4,382 | 6.2 (4.7-8.0) | 4.0 (2.8-5.8) | 3.5 (2.3-5.2) | 2.2 (1.3-3.9) | 2.4 (1.5-4.0) | 3.8 | <0.001 |
| Zambia 2018 | 8,694 | 4.4 (3.4-5.6) | 4.2 (3.3-5.5) | 2.9 (2.1-3.9) | 5.3 (4.0-7.1) | 6.0 (3.8-9.2) | -1.6 | 0.067 |
| Zimbabwe 2015 | 4,897 | 4.6 (3.4-6.1) | 4.9 (3.6-6.6) | 3.2 (2.2-4.8) | 3.7 (2.6-5.2) | 1.7 (0.9-3.3) | 2.9 | 0.017 |

Q1, poorest quintile; Q2, poorer quintile; Q3, middle quintile; Q4, richer quintile; Q5, richest quintile.
