# Supplementary material for: Socioeconomic and urban-rural inequalities in the population-level double burden of child malnutrition in the East and Southern African Region
Source: PLOS Glob Public Health. 2023 Apr 25;3(4):e0000397. doi: 10.1371/journal.pgph.0000397 (PMC10128925; doi:10.1371/journal.pgph.0000397)
Supplement: S10 Table — (DOCX) [file pgph.0000397.s010.docx]

**S10 Table**. Maternal education differentials in child wasting by country and year

|  |  | Education level | | | | | |
| --- | --- | --- | --- | --- | --- | --- | --- |
| **Country and survey year** | **Sample size** | **E1**  **(95% CI)** | **E2**  **(95% CI)** | **E3**  **(95% CI)** | **E4**  **(95% CI)** | **Gap**  **(% points)** | **p-value**  **(E1-E4)** |
| Comoros 2012 | 2,432 | 13.8 (11.5-16.5) | 8.6 (6.1-11.9) | 10.5 (7.9-13.8) | 11.9 (6.4-21.0) | 1.9 | 0.066 |
| Eswatini 2006 | 2,042 | 1.6 (0.5-5.0) | 3.5 (2.4-5.1) | 2.4 (1.5-3.7) | 1.1 (0.3-4.9) | 0.5 | 0.251 |
| Kenya 2014 | 18,648 | 10.5 (8.9-12.5) | 3.5 (3.1-4.1) | 3.4 (2.7-4.2) | 1.9 (1.1-3.1) | 8.6 | <0.001 |
| Lesotho 2014 | 1,303 | ND | 5.2 (3.7-7.3) | 2.0 (1.1-3.5) | 0.3 (0.0-1.9) | ND | 0.002 |
| Malawi 2015-16 | 5,116 | 2.3 (1.4-3.7) | 2.9 (2.4-3.7) | 2.9 (1.9-4.5) | 5.5 (1.3-20.6) | -3.2 | 0.567 |
| Mozambique 2011 | 9,363 | 7.6 (6.6-8.8) | 6.0 (5.1-7.0) | 3.6 (2.5-5.2) | ND | ND | ND |
| Namibia 2013 | 1,800 | 17.1 (11.9-23.8) | 8.2 (5.9-11.2) | 8.3 (6.4-10.6) | 0.9 (0.2-3.8) | 16.2 | <0.001 |
| Rwanda 2014-15 | 3,544 | 2.9 (1.7-4.9) | 2.2 (1.7-2.8) | 2.5 (1.3-4.6) | 1.9 (0.5-7.5) | 1.0 | 0.754 |
| South Africa 2016 | 1,070 | 3.4 (0.4-21.3) | 1.6 (0.5-4.8) | 2.9 (1.7-4.8) | 0.7 (0.1-5.0) | 2.7 | 0.390 |
| Tanzania 2015-16 | 8,940 | 5.4 (4.3-6.7) | 4.9 (4.3-5.7) | 3.9 (2.8-5.3) | 2.5 (0.6-9.7) | 2.9 | 0.318 |
| Uganda 2016 | 4,382 | 4.6 (2.9-7.2) | 4.0 (3.2-4.9) | 2.8 (1.7-4.6) | 3.8 (1.8-8.0) | 0.8 | 0.486 |
| Zambia 2018 | 8,694 | 4.5 (2.8-7.0) | 4.0 (3.3-4.8) | 5.2 (4.2-6.4) | 4.9 (2.5-9.1) | 0.4 | 0.270 |
| Zimbabwe 2015 | 4,897 | 3.4 (0.8-13.2) | 4.8 (3.8-6.2) | 3.3 (2.6-4.1) | 3.1 (1.3-6.9) | 0.3 | 0.123 |

E1, no education; E2, primary education; E3, secondary education; E4, higher education.
ND = insufficient data collected
