# Supplementary material for: Socioeconomic and urban-rural inequalities in the population-level double burden of child malnutrition in the East and Southern African Region
Source: PLOS Glob Public Health. 2023 Apr 25;3(4):e0000397. doi: 10.1371/journal.pgph.0000397 (PMC10128925; doi:10.1371/journal.pgph.0000397)
Supplement: S11 Table — (DOCX) [file pgph.0000397.s011.docx]

**S11 Table.** Residence differentials in child wasting by country and year

|  | | Area of residence | | | |
| --- | --- | --- | --- | --- | --- |
| **Country and survey year** | **Sample size** | **Rural**  **(95% CI)** | **Urban**  **(95% CI)** | **Gap**  **(% points)** | **p-value**  **(rural-urban)** |
| Comoros 2012 | 2,432 | 11.0 (9.3-12.9) | 13.8 (10.7-17.6) | 2.8 | 0.133 |
| Eswatini 2006 | 2,042 | 2.6 (1.9-3.5) | 2.9 (1.7-4.9) | -0.3 | 0.743 |
| Kenya 2014 | 18,648 | 4.5 (4.0-5.2) | 3.6 (3.0-4.3) | 0.9 | 0.040 |
| Lesotho 2014 | 1,303 | 4.0 (2.9-5.5) | 1.7 (0.7-3.9) | 2.3 | 0.051 |
| Malawi 2015-16 | 5,116 | 2.9 (2.3-3.5) | 3.2 (1.8-5.6) | -0.3 | 0.685 |
| Mozambique 2011 | 9,363 | 7.1 (6.3-8.1) | 4.1 (3.3-5.1) | 3.0 | <0.001 |
| Namibia 2013 | 1,800 | 8.8 (7.1-10.7) | 8.0 (5.6-11.3) | 0.8 | 0.671 |
| Rwanda 2014-15 | 3,544 | 2.4 (1.9-3.0) | 2.0 (1.2-3.4) | 0.4 | 0.598 |
| South Africa 2016 | 1,070 | 3.3 (1.8-5.9) | 2.0 (1.0-4.1) | 1.3 | 0.302 |
| Tanzania 2015-16 | 8,940 | 5.2 (4.6-5.9) | 4.0 (3.0-5.2) | 1.2 | 0.075 |
| Uganda 2016 | 4,382 | 4.0 (3.3-4.8) | 3.0 (1.9-4.6) | 1.0 | 0.238 |
| Zambia 2018 | 8,694 | 4.0 (3.4-4.8) | 5.4 (4.1-7.1) | -1.4 | 0.082 |
| Zimbabwe 2015 | 4,897 | 4.1 (3.4-5.0) | 2.8 (2.0-4.1) | 1.3 | 0.079 |
