# Supplementary material for: Socioeconomic and urban-rural inequalities in the population-level double burden of child malnutrition in the East and Southern African Region
Source: PLOS Glob Public Health. 2023 Apr 25;3(4):e0000397. doi: 10.1371/journal.pgph.0000397 (PMC10128925; doi:10.1371/journal.pgph.0000397)
Supplement: S12 Table — (DOCX) [file pgph.0000397.s012.docx]

**S12 Table.** Wealth index differentials of child overweight (including obesity) by country and year

|  |  | Wealth index | | | | | | |
| --- | --- | --- | --- | --- | --- | --- | --- | --- |
| **Country and survey year** | **Sample size** | **Q1**  **(95% CI)** | **Q2**  **(95% CI)** | **Q3**  **(95% CI)** | **Q4**  **(95% CI)** | **Q5**  **(95% CI)** | **Gap**  **(% points)** | **p-value**  **(Q1-Q5)** |
| Comoros 2012 | 2,432 | 7.4 (5.2-10.5) | 9.6 (6.5-13.9) | 9.7 (6.9-13.3) | 13.5 (9.5-19.0) | 10.5 (7.0-15.3) | -3.1 | 0.192 |
| Eswatini 2006 | 2,042 | 8.8 (6.3-12.1) | 8.6 (6.3-11.6) | 12.6 (8.8-17.6) | 11.4 (8.4-15.3) | 14.9 (11.7-18.8) | -6.1 | 0.058 |
| Kenya 2014 | 18,648 | 2.2 (1.8-2.8) | 3.5 (2.9-4.2) | 4.2 (3.4-5.3) | 5.5 (4.4-6.9) | 6.4 (5.1-8.0) | -4.2 | <0.001 |
| Lesotho 2014 | 1,303 | 9.1 (6.1-13.3) | 7.4 (4.8-11.4) | 7.9 (4.8-12.7) | 7.2 (4.2-12.1) | 8.1 (4.1-15.2) | 1.0 | 0.961 |
| Malawi 2015-16 | 5,116 | 3.5 (2.4-5.2) | 4.9 (3.5-7.0) | 4.9 (3.5-6.7) | 4.6 (3.3-6.5) | 4.8 (3.2-7.0) | -1.3 | 0.656 |
| Mozambique 2011 | 9,363 | 5.7 (4.5-7.2) | 7.3 (5.7-9.2) | 8.3 (6.7-10.1) | 8.6 (7.1-10.3) | 8.6 (7.2-10.3) | -2.9 | 0.036 |
| Namibia 2013 | 1,800 | 2.3 (1.2-4.3) | 4.8 (3.0-7.6) | 3.9 (2.3-6.6) | 6.4 (4.1-9.9) | 5.6 (2.8-10.9) | -3.3 | 0.107 |
| Rwanda 2014-15 | 3,544 | 7.3 (5.6-9.4) | 7.8 (6.0-10.0) | 6.3 (4.7-8.6) | 9.1 (6.9-11.8) | 11.0 (8.6-13.9) | -3.7 | 0.043 |
| South Africa 2016 | 1,070 | 15.5 (9.7-23.9) | 14.4 (9.7-20.9) | 10.9 (6.8-16.9) | 14.6 (9.6-21.4) | 11.1 (4.8-23.8) | 4.4 | 0.776 |
| Tanzania 2015-16 | 8,940 | 2.8 (2.1-3.6) | 3.3 (2.3-4.6) | 4.1 (3.1-5.4) | 3.9 (2.9-5.3) | 5.3 (4.0-6.8) | -2.5 | 0.029 |
| Uganda 2016 | 4,382 | 3.0 (1.8-5.1) | 4.4 (3.1-6.3) | 4.6 (3.3-6.4) | 4.6 (3.1-6.8) | 3.4 (2.2-5.3) | -0.4 | 0.516 |
| Zambia 2018 | 8,694 | 5.7 (4.6-7.0) | 4.3 (3.4-5.5) | 4.8 (3.7-6.1) | 5.6 (4.0-7.7) | 5.6 (4.1-7.6) | 0.1 | 0.473 |
| Zimbabwe 2015 | 4,897 | 5.2 (3.9-7.0) | 4.9 (3.5-6.9) | 5.5 (4.0-7.4) | 7.0 (5.5-8.8) | 7.5 (5.6-10.0) | -2.3 | 0.159 |

Q1, poorest quintile; Q2, poorer quintile; Q3, middle quintile; Q4, richer quintile; Q5, richest quintile.
