# Supplementary material for: Socioeconomic and urban-rural inequalities in the population-level double burden of child malnutrition in the East and Southern African Region
Source: PLOS Glob Public Health. 2023 Apr 25;3(4):e0000397. doi: 10.1371/journal.pgph.0000397 (PMC10128925; doi:10.1371/journal.pgph.0000397)
Supplement: S13 Table — (DOCX) [file pgph.0000397.s013.docx]

**S13 Table.** Maternal education differentials of child overweight (including obesity) by country and year

|  | | Maternal education level | | | | | |
| --- | --- | --- | --- | --- | --- | --- | --- |
| **Country and survey year** | **Sample size** | **E1**  **(95% CI)** | **E2**  **(95% CI)** | **E3**  **(95% CI)** | **E4**  **(95% CI)** | **Gap**  **(% points)** | **p-value**  **(E1-E4)** |
| Comoros 2012 | 2,432 | 8.3 (6.3-10.8) | 13.3 (10.2-17.2) | 9.9 (6.9-13.9) | 9.3 (5.0-16.5) | -1.0 | 0.088 |
| Eswatini 2006 | 2,042 | 7.7 (4.7-12.2) | 8.6 (6.6-11.1) | 12.3 (10.1-15.0) | 19.9 (14.0-27.3) | -12.2 | 0.001 |
| Kenya 2014 | 18,648 | 2.2 (1.6-3.0) | 3.5 (3.0-4.0) | 5.7 (4.7-6.9) | 8.4 (6.4-11.0) | -6.2 | <0.001 |
| Lesotho 2014 | 1,303 | 14.6 (3.3-45.8) | 6.6 (4.8-9.0) | 7.5 (5.4-10.4) | 18.6 (10.2-31.6) | -4.0 | 0.008 |
| Malawi 2015-16 | 5,116 | 4.0 (2.2-7.1) | 4.6 (3.7-5.6) | 4.2 (2.9-6.1) | 7.6 (3.4-16.3) | -3.6 | 0.659 |
| Mozambique 2011 | 9,363 | 6.8 (5.9-7.9) | 7.6 (6.4-8.8) | 8.9 (7.9-12.3) | 11.0 (5.7-20.0) | -5.2 | 0.056 |
| Namibia 2013 | 1,800 | 2.8 (1.0-7.6) | 3.0 (1.8-5.0) | 4.8 (3.6-6.5) | 8.5 (3.2-21.0) | -5.7 | 0.156 |
| Rwanda 2014-15 | 3,544 | 7.3 (5.2-10.3) | 7.9 (6.9-9.0) | 10.1 (7.2-13.9) | 11.2 (5.9-20.3) | -3.9 | 0.361 |
| South Africa 2016 | 1,070 | ND | 20.1 (8.8-39.6) | 13.4 (10.8-16.5) | 10.3 (5.0-20.2) | ND | 0.312 |
| Tanzania 2015-16 | 8,940 | 2.5 (1.8-3.5) | 3.7 (3.1-4.4) | 5.9 (4.5-7.7) | 5.0 (1.4-16.3) | -2.5 | 0.002 |
| Uganda 2016 | 4,382 | 2.2 (1.3-3.8) | 3.9 (3.1-4.9) | 4.7 (3.2-7.0) | 5.0 (2.7-9.2) | -2.8 | 0.223 |
| Zambia 2018 | 8,694 | 5.2 (3.6-7.5) | 5.1 (4.4-6.0) | 5.2 (4.3-6.2) | 6.2 (3.6-10.4) | -1.0 | 0.924 |
| Zimbabwe 2015 | 4,897 | 3.4 (0.9-11.6) | 5.0 (3.9-7.2) | 6.2 (5.3-7.2) | 9.9 (6.3-15.4) | -6.5 | 0.024 |

E1, no education; E2, primary education; E3, secondary education; E4, higher education.
