# Supplementary material for: Socioeconomic and urban-rural inequalities in the population-level double burden of child malnutrition in the East and Southern African Region
Source: PLOS Glob Public Health. 2023 Apr 25;3(4):e0000397. doi: 10.1371/journal.pgph.0000397 (PMC10128925; doi:10.1371/journal.pgph.0000397)
Supplement: S14 Table — (DOCX) [file pgph.0000397.s014.docx]

**S14 Table.** Residence differentials of child overweight (including obesity) by country and year

|  | | Area of residence | | | |
| --- | --- | --- | --- | --- | --- |
| **Country and survey year** | **Sample size** | **Rural**  **(95% CI)** | **Urban**  **(95% CI)** | **Gap**  **(% points)** | **p-value**  **(rural-urban)** |
| Comoros 2012 | 2,432 | 9.6 (7.8-11.7) | 10.6 (8.0-14.0) | -1.0 | 0.568 |
| Eswatini 2006 | 2,042 | 9.7 (8.3-11.3) | 16.5 (12.3-21.9) | -6.8 | 0.002 |
| Kenya 2014 | 18,648 | 3.6 (3.2-4.0) | 5.5 (4.6-6.6) | -1.9 | <0.001 |
| Lesotho 2014 | 1,303 | 8.0 (6.4-10.1) | 7.7 (4.9-11.9) | 0.3 | 0.843 |
| Malawi 2015-16 | 5,116 | 4.5 (3.8-5.4) | 4.5 (2.9-6.7) | 0.0 | 0.953 |
| Mozambique 2011 | 9,363 | 7.2 (6.4-8.1) | 8.6 (7.3-10.1) | -1.3 | 0.095 |
| Namibia 2013 | 1,800 | 4.1 (2.9-5.6) | 5.0 (3.4-7.3) | -0.9 | 0.419 |
| Rwanda 2014-15 | 3,544 | 7.5 (6.6-8.6) | 11.3 (9.0-14.0) | -3.8 | 0.003 |
| South Africa 2016 | 1,070 | 12.7 (9.7-16.4) | 14.1 (10.4-18.7) | -1.4 | 0.618 |
| Tanzania 2015-16 | 8,940 | 3.7 (3.1-4.3) | 4.0 (3.1-5.2) | -0.3 | 0.574 |
| Uganda 2016 | 4,382 | 4.2 (3.4-5.0) | 3.2 (2.1-4.8) | 1.0 | 0.249 |
| Zambia 2018 | 8,694 | 5.0 (4.3-5.7) | 5.6 (4.5-7.1) | -0.6 | 0.351 |
| Zimbabwe 2015 | 4,897 | 5.2 (4.3-6.2) | 7.9 (6.4-9.7) | -2.7 | 0.002 |
