# Supplementary material for: Socioeconomic and urban-rural inequalities in the population-level double burden of child malnutrition in the East and Southern African Region
Source: PLOS Glob Public Health. 2023 Apr 25;3(4):e0000397. doi: 10.1371/journal.pgph.0000397 (PMC10128925; doi:10.1371/journal.pgph.0000397)
Supplement: S15 Table — (DOCX) [file pgph.0000397.s015.docx]

**S15 Table**. Country-specific household wealth gradient of stunting among children under five — slope index of inequality (SII) and relative index of inequality (RII) on magnitude of inequality in stunting

| Country | SII | RII |
| --- | --- | --- |
| Comoros 2012 | -0.18 (-0.24,-0.11) | 0.53(0.41,0.65) |
| Eswatini 2006 | -0.24(-0.31,-0.17) | 0.40(0.30,0.51) |
| Kenya 2015 | -0.25(-0.27,-0.23) | 0.39(0.35,0.42) |
| Lesotho 2014 | -0.32(-0.40,-0.24) | 0.38(0.28,0.48) |
| Malawi 2015 | -0.22(-0.27,-0.18) | 0.52(0.45,0.59) |
| Mozambique 2011 | -0.31(-0.35,-0.28) | 0.44(0.40,0.48) |
| Namibia 2013 | -0.22(-0.29,-0.16) | 0.36(0.25,0.47) |
| Rwanda 2014 | -0.36(-0.41,-0.31) | 0.37(0.31,0.42) |
| South Africa 2016 | -0.24(-0.32,-0.15) | 0.38(0.23,0.52) |
| Tanzania 2015 | -0.21(-0.24,-0.18) | 0.53(0.47,0.58) |
| Uganda 2016 | -0.18(-0.22,-0.13) | 0.53(0.45,0.62) |
| Zambia 2018 | -0.18(-0.21,-0.15) | 0.59(0.53,0.65) |
| Zimbabwe 2015 | -0.18(-0.22,-0.14) | 0.49(0.41,0.58) |
