# Supplementary material for: Socioeconomic and urban-rural inequalities in the population-level double burden of child malnutrition in the East and Southern African Region
Source: PLOS Glob Public Health. 2023 Apr 25;3(4):e0000397. doi: 10.1371/journal.pgph.0000397 (PMC10128925; doi:10.1371/journal.pgph.0000397)
Supplement: S16 Table — (DOCX) [file pgph.0000397.s016.docx]

**S16 Table**. Country-specific maternal education gradient of stunting among children under five — slope index of inequality (SII) and relative index of inequality (RII) on magnitude of inequality in stunting

| Country | SII | RII |
| --- | --- | --- |
| Comoros 2012 | -0.16(-0.22,-0.09) | 0.57(0.44,0.70) |
| Eswatini 2006 | -0.25(-0.31,-0.18) | 0.40(0.29,0.50) |
| Kenya 2015 | -0.15(-0.18,-0.13) | 0.56(0.52,0.61) |
| Lesotho 2014 | -0.26(-0.36,-0.17) | 0.46(0.32,0.59) |
| Malawi 2015 | -0.19(-0.24,-0.13) | 0.58(0.49,0.67) |
| Mozambique 2011 | -0.22(-0.25,-0.18) | 0.57(0.52,0.63) |
| Namibia 2013 | -0.24(-0.32,-0.17) | 0.33(0.22,0.43) |
| Rwanda 2014 | -0.33(-0.39,-0.26) | 0.41(0.33,0.48) |
| South Africa 2016 | -0.30(-0.42,-0.19) | 0.28(0.15,0.42) |
| Tanzania 2015 | -0.18(-0.22,-0.15) | 0.58(0.51,0.64) |
| Uganda 2016 | -0.22(-0.27,-0.17) | 0.46(0.37,0.54) |
| Zambia 2018 | -0.17(-0.21,-0.13) | 0.61(0.54,0.68) |
| Zimbabwe 2015 | -0.19(-0.23,-0.14) | 0.48(0.39,0.57) |
