# Supplementary material for: Socioeconomic and urban-rural inequalities in the population-level double burden of child malnutrition in the East and Southern African Region
Source: PLOS Glob Public Health. 2023 Apr 25;3(4):e0000397. doi: 10.1371/journal.pgph.0000397 (PMC10128925; doi:10.1371/journal.pgph.0000397)
Supplement: S17 Table — (DOCX) [file pgph.0000397.s017.docx]

**S17 Table**. Country-specific household wealth gradient of wasting among children under five — slope index of inequality (SII) and relative index of inequality (RII) on magnitude of inequality in wasting

| Country | SII | RII |
| --- | --- | --- |
| Comoros 2012 | -0.02(-0.07,0.02) | 0.82(0.51,1.13) |
| Eswatini 2006 | -0.02(-0.04,0.01) | 0.54(0.06-1.03) |
| Kenya 2015 | -0.10(-0.12,-0.09) | 0.17(0.13,0.21) |
| Lesotho 2014 | -0.05(-0.09,-0.01) | 0.23(0.00,0.46) |
| Malawi 2015 | -0.01(-0.03,0.01) | 0.75(0.35,1.16) |
| Mozambique 2011 | -0.08(-0.10,-0.06) | 0.22(0.15,0.30) |
| Namibia 2013 | -0.05(-0.09,0.00) | 0.58(0.28,0.89) |
| Rwanda 2014 | -0.01(-0.03,0.01) | 0.60(0.17,1.04) |
| South Africa 2016 | 0.00(-0.03,0.03) | 0.97(-0.05,2.00) |
| Tanzania 2015 | 0.00(-0.02,0.01) | 0.97(0.67,1.27) |
| Uganda 2016 | -0.06(-0.08,-0.03) | 0.22(0.09,0.36) |
| Zambia 2018 | 0.00(-0.01,0.02) | 1.07(0.67,1.46) |
| Zimbabwe 2015 | -0.04(-0.05,-0.02) | 0.38(0.19,0.57) |
