# Supplementary material for: Socioeconomic and urban-rural inequalities in the population-level double burden of child malnutrition in the East and Southern African Region
Source: PLOS Glob Public Health. 2023 Apr 25;3(4):e0000397. doi: 10.1371/journal.pgph.0000397 (PMC10128925; doi:10.1371/journal.pgph.0000397)
Supplement: S18 Table — (DOCX) [file pgph.0000397.s018.docx]

**S18 Table**. Country-specific maternal education gradient of wasting among children under five — slope index of inequality (SII) and relative index of inequality (RII) on magnitude of inequality in wasting

| Country | SII | RII |
| --- | --- | --- |
| Comoros 2012 | -0.04(-0.09,0.01) | 0.71(0.42,1.00) |
| Eswatini 2006 | -0.01(-0.03,0.01) | 0.69(0.05,1.34) |
| Kenya 2015 | -0.13(-0.15,-0.11) | 0.10(0.08,0.13) |
| Lesotho 2014 | -0.04(-0.08,0.00) | 0.36(-0.02,0.73) |
| Malawi 2015 | 0.01(-0.01,0.03) | 1.20(0.47,1.92) |
| Mozambique 2011 | -0.06(-0.07,-0.04) | 0.36(0.25,0.48) |
| Namibia 2013 | -0.07(-0.12,-0.02) | 0.45(0.18,0.71) |
| Rwanda 2014 | 0.00(-0.02,0.02) | 0.99(-0.01,1.99) |
| South Africa 2016 | -0.02(-0.06,0.02) | 0.53(-0.20,1.26) |
| Tanzania 2015 | 0.00(-0.02,0.01) | 0.91(0.58,1.24) |
| Uganda 2016 | -0.03(-0.05,0.00) | 0.47(0.18,0.76) |
| Zambia 2018 | 0.01(-0.01,0.02) | 1.20(0.74,1.66) |
| Zimbabwe 2015 | -0.03(-0.05,-0.01) | 0.43(0.19,0.68) |
