# Supplementary material for: Socioeconomic and urban-rural inequalities in the population-level double burden of child malnutrition in the East and Southern African Region
Source: PLOS Glob Public Health. 2023 Apr 25;3(4):e0000397. doi: 10.1371/journal.pgph.0000397 (PMC10128925; doi:10.1371/journal.pgph.0000397)
Supplement: S19 Table — (DOCX) [file pgph.0000397.s019.docx]

**S19 Table**. Country-specific household wealth gradient of overweight (including obesity) among children under five— slope index of inequality (SII) and relative index of inequality (RII) on magnitude of inequality in overweight (including obesity)

| Country | SII | RII |
| --- | --- | --- |
| Comoros 2012 | 0.09(0.04,0.13) | 2.45(1.38,3.52) |
| Eswatini 2006 | 0.07(0.02,0.12) | 1.91(1.05,2.77) |
| Kenya 2015 | 0.05(0.04,0.06) | 4.46(3.24,5.67) |
| Lesotho 2014 | 0.00(-0.06,0.05) | 0.97(0.33,1.61) |
| Malawi 2015 | 0.01(-0.01,0.03) | 1.33(0.73,1.93) |
| Mozambique 2011 | 0.04(0.02,0.05) | 1.57(1.19,1.95) |
| Namibia 2013 | 0.04(0.00,0.07) | 2.34(0.59,4.09) |
| Rwanda 2014 | 0.04(0.01,0.07) | 1.65(0.98,2.32) |
| South Africa 2016 | -0.01(-0.08,0.06) | 0.94(0.42,1.46) |
| Tanzania 2015 | 0.02(0.00,0.03) | 1.66(1.01,2.32) |
| Uganda 2016 | 0.02(0.00,0.04) | 1.70(0.88,2.51) |
| Zambia 2018 | 0.01(-0.01,0.02) | 1.12(0.75,1.49) |
| Zimbabwe 2015 | 0.03(0.01,0.06) | 1.64(1.00,2.29) |
